# 4-[(*E*)-Phenyl­imino­meth­yl]benzonitrile

**DOI:** 10.1107/S1600536808008672

**Published:** 2008-04-02

**Authors:** Mmuhammad Akram Kashmiri, M. Nawaz Tahir, Muhammad Akhtar, Mushtaq Ahmed, Asif Hanif Ch

**Affiliations:** a Government College University, Department of Chemistry, Lahore, Pakistan; b University of Sargodha, Department of Physics, Sargodha, Pakistan

## Abstract

In the mol­ecule of the title compound, C_14_H_10_N_2_, the two aromatic rings are oriented at a dihedral angle of 32.22 (6)°. In the crystal structure, inter­molecular C—H⋯N hydrogen bonds link the mol­ecules into centrosymmetric *R*_2_^2^(10) dimers. A weak π–π inter­action between the cyanobenzene rings, with a centroid–centroid distance of 3.8447 (3) Å, further stabilizes the crystal structure. There is also a C—H⋯π inter­action between the aniline ring and a CH group of the cyanobenzene ring.

## Related literature

For related structures, see: Ojala *et al.* (2002 ▶). For ring motif details, see: Bernstein *et al.* (1995 ▶); Etter (1990 ▶). For bond-length data, see: Allen *et al.* (1987 ▶).
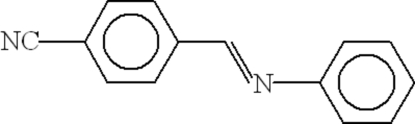


## Experimental

### 

#### Crystal data

C_14_H_10_N_2_
*M* *_r_* = 206.24
Monoclinic, 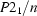

*a* = 7.2673 (4) Å
*b* = 10.0287 (7) Å
*c* = 15.4306 (12) Å
β = 96.177 (2)°
*V* = 1118.08 (13) Å^3^
*Z* = 4
Mo *K*α radiation
μ = 0.07 mm^−1^
*T* = 296 (2) K
0.20 × 0.15 × 0.12 mm

#### Data collection

Bruker Kappa-APEXII CCD diffractometer
Absorption correction: multi-scan (*SADABS*; Bruker, 2005 ▶) *T*_min_ = 0.983, *T*_max_ = 0.994
13128 measured reflections
2883 independent reflections
1423 reflections with *I* > 2σ(*I*)
*R*_int_ = 0.047

#### Refinement

*R*[*F* ^2^ > 2σ(*F* ^2^)] = 0.046
*wR*(*F* ^2^) = 0.118
*S* = 1.03
2883 reflections
176 parameters
All H-atom parameters refined
Δρ_max_ = 0.13 e Å^−3^
Δρ_min_ = −0.10 e Å^−3^

### 

Data collection: *APEX2* (Bruker, 2007 ▶); cell refinement: *APEX2*; data reduction: *SAINT* (Bruker, 2007 ▶); program(s) used to solve structure: *SHELXS97* (Sheldrick, 2008 ▶); program(s) used to refine structure: *SHELXL97* (Sheldrick, 2008 ▶); molecular graphics: *ORTEP-3 for Windows* (Farrugia, 1997 ▶) and *PLATON* (Spek, 2003 ▶); software used to prepare material for publication: *WinGX* (Farrugia, 1999 ▶) and *PLATON*.

## Supplementary Material

Crystal structure: contains datablocks global, I. DOI: 10.1107/S1600536808008672/hk2445sup1.cif

Structure factors: contains datablocks I. DOI: 10.1107/S1600536808008672/hk2445Isup2.hkl

Additional supplementary materials:  crystallographic information; 3D view; checkCIF report

## Figures and Tables

**Table 1:** Hydrogen-bond geometry (Å, °)

*D*—H⋯*A*	*D*—H	H⋯*A*	*D*⋯*A*	*D*—H⋯*A*
C3—H3⋯N2^i^	0.980 (13)	2.616 (14)	3.473 (2)	146.1 (10)
C5—H5⋯*Cg*^ii^	0.975 (13)	2.650 (14)	3.5970 (17)	163.9 (11)

Symmetry codes: (i) 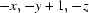; (ii) 
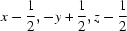
. *Cg* is the centroid of atoms C9–C14.

**Fig. 1.**
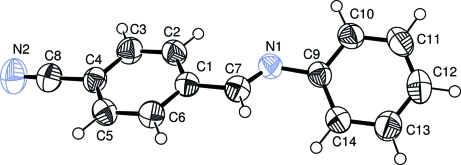
The molecular structure of the title molecule, with the atom-numbering scheme. Displacement ellipsoids are drawn at the 50% probability level.

**Fig. 2.**
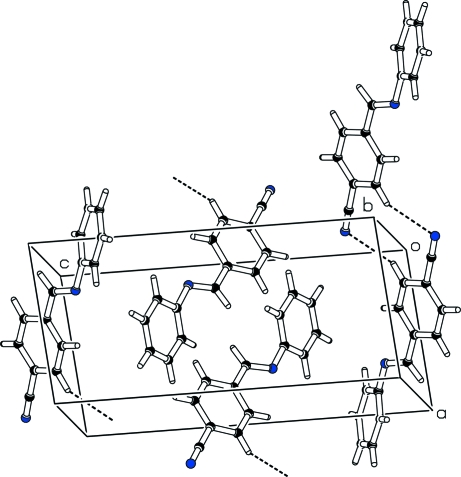
A partial packing diagram of (I), showing the formation of centro- symmetric R22(10) ring motifs. Hydrogen bonds are shown as dashed lines.

## supplementary crystallographic information

### Comment

The crystal structures of *p*-halo-*N*-(*p*-cyanobenzylidene)- aniline and *p*-cyano-*N*-(*p*-halobenzylidene)aniline, (halo = bromo and chloro) (Ojala *et al.*,  2002) have been reported, previously. The title compound, (I), differs due to no attachment of halogen atoms. It is prepared in aqueous medium and we report herein its crystal structure.

The molecule of (I), (Fig. 1), is a Schiff base ligand of aniline and *p*-cyanobenzaldehyde. The bond lengths (Allen *et al.*, 1987) and angles are generally within normal ranges. Rings A (C1-C6) and B (C9-C14) are, of course, planar, and they are oriented at a dihedral angle of 32.22 (6)°.

In the crystal structure, intermolecular C-H···N hydrogen bonds (Table 1) link the molecules into centrosymmetric R_2_^2^(10) dimers (Fig. 2) (Bernstein *et al.*,  1995; Etter, 1990), in which they may be effective in the stabilization of the structure. A weak π···π interaction between the A rings, at x, y, z and 1 - x, 1 - y, -z, further stabilizes the structure, with a centroid-centroid distance of 3.8447 (3) Å. There is also a C—H···π interaction between the ring B at  x - 1/2, 1/2 -y, z - 1/2 and C5-H5, with H5-centroid distance of 2.650 (14) Å.

### Experimental

The starting materials employed were first purified by distillation or crystallization just before use. The experiment was performed in stoppered flask at room temperature. The title compound was synthesized by using equimolecular mixture of aniline (5 mmol) and *p*-cyanobenzaldehyde (5 mmol) of pH = 9 in aqueous medium. The product was precipitated after a few minutes, and separated by filtration, washed with a small amount of water and dried for 2 d at room temperature in a vacuum desicator. The dried filtrate was used for X-ray analysis (yield; 53.09%, m.p. 339 K).

### Refinement

H atoms were located in a difference syntheses and refined [C-H = 0.949 (15)-0.999 (12) Å and U_iso_(H) = 1.2U_eq_(C)].

### Crystal data

**Table:**

C_14_H_10_N_2_	*F*_000_ = 432
*M**_r_* = 206.24	*D*_x_ = 1.226 Mg m^−^^3^
Monoclinic, *P*2_1_/*n*	Mo *K*α radiation λ = 0.71073 Å
Hall symbol: -P 2yn	Cell parameters from 2883 reflections
*a* = 7.2673 (4) Å	θ = 2.4–28.7º
*b* = 10.0287 (7) Å	µ = 0.07 mm^−^^1^
*c* = 15.4306 (12) Å	*T* = 296 (2) K
β = 96.177 (2)º	Prismatic, yellow
*V* = 1118.08 (13) Å^3^	0.20 × 0.15 × 0.12 mm
*Z* = 4	

### Data collection

**Table:**

Bruker Kappa-APEXII CCD diffractometer	2883 independent reflections
Radiation source: fine-focus sealed tube	1423 reflections with *I* > 2σ(*I*)
Monochromator: graphite	*R*_int_ = 0.047
Detector resolution: 7.30 pixels mm^-1^	θ_max_ = 28.7º
*T* = 296(2) K	θ_min_ = 2.4º
ω scans	*h* = −9→9
Absorption correction: multi-scan(SADABS; Bruker, 2005)	*k* = −13→13
*T*_min_ = 0.983, *T*_max_ = 0.994	*l* = −20→20
13128 measured reflections	

### Refinement

**Table:**

Refinement on *F*^2^	Secondary atom site location: difference Fourier map
Least-squares matrix: full	Hydrogen site location: inferred from neighbouring sites
*R*[*F*^2^ > 2σ(*F*^2^)] = 0.046	All H-atom parameters refined
*wR*(*F*^2^) = 0.118	*w* = 1/[σ^2^(*F*_o_^2^) + (0.0364*P*)^2^] where *P* = (*F*_o_^2^ + 2*F*_c_^2^)/3
*S* = 1.03	(Δ/σ)_max_ < 0.001
2883 reflections	Δρ_max_ = 0.13 e Å^−^^3^
176 parameters	Δρ_min_ = −0.10 e Å^−^^3^
Primary atom site location: structure-invariant direct methods	Extinction correction: none

### Special details

**Table:**

Geometry. All e.s.d.'s (except the e.s.d. in the dihedral angle between two l.s. planes) are estimated using the full covariance matrix. The cell e.s.d.'s are taken into account individually in the estimation of e.s.d.'s in distances, angles and torsion angles; correlations between e.s.d.'s in cell parameters are only used when they are defined by crystal symmetry. An approximate (isotropic) treatment of cell e.s.d.'s is used for estimating e.s.d.'s involving l.s. planes.
Refinement. Refinement of F^2^ against ALL reflections. The weighted R-factor wR and goodness of fit S are based on F^2^, conventional R-factors R are based on F, with F set to zero for negative F^2^. The threshold expression of F^2^ > 2sigma(F^2^) is used only for calculating R-factors(gt) etc. and is not relevant to the choice of reflections for refinement. R-factors based on F^2^ are statistically about twice as large as those based on F, and R- factors based on ALL data will be even larger.

### Fractional atomic coordinates and isotropic or equivalent isotropic displacement parameters (Å^2^)

**Table:**

	*x*	*y*	*z*	*U*_iso_*/*U*_eq_	
N1	0.70615 (14)	0.08086 (11)	0.10666 (8)	0.0518 (3)	
N2	0.05182 (19)	0.57458 (14)	−0.13327 (10)	0.0855 (5)	
C1	0.57569 (17)	0.23345 (13)	−0.00193 (9)	0.0462 (4)	
C2	0.40486 (19)	0.23930 (15)	0.03050 (10)	0.0559 (4)	
H2	0.3870 (17)	0.1816 (14)	0.0777 (9)	0.067*	
C3	0.27073 (19)	0.32560 (16)	−0.00409 (10)	0.0593 (4)	
H3	0.1494 (18)	0.3263 (13)	0.0181 (9)	0.071*	
C4	0.30456 (17)	0.40866 (13)	−0.07259 (9)	0.0493 (4)	
C5	0.4727 (2)	0.40351 (15)	−0.10663 (10)	0.0538 (4)	
H5	0.4929 (16)	0.4628 (13)	−0.1547 (9)	0.065*	
C6	0.60682 (19)	0.31589 (15)	−0.07083 (10)	0.0528 (4)	
H6	0.7273 (17)	0.3117 (13)	−0.0919 (9)	0.063*	
C7	0.72303 (19)	0.14630 (14)	0.03760 (10)	0.0519 (4)	
H7	0.8385 (17)	0.1419 (12)	0.0080 (8)	0.062*	
C8	0.1639 (2)	0.50057 (16)	−0.10713 (10)	0.0610 (4)	
C9	0.85826 (18)	0.00574 (13)	0.14575 (9)	0.0476 (4)	
C10	0.8196 (2)	−0.11183 (15)	0.18708 (10)	0.0569 (4)	
H10	0.6901 (18)	−0.1361 (13)	0.1860 (9)	0.068*	
C11	0.9612 (2)	−0.18768 (16)	0.22793 (10)	0.0657 (5)	
H11	0.9274 (18)	−0.2675 (15)	0.2552 (10)	0.079*	
C12	1.1415 (2)	−0.14576 (17)	0.22995 (11)	0.0668 (5)	
H12	1.2406 (18)	−0.1973 (15)	0.2609 (10)	0.080*	
C13	1.1803 (2)	−0.02736 (18)	0.19159 (11)	0.0684 (5)	
H13	1.305 (2)	0.0051 (15)	0.1918 (9)	0.082*	
C14	1.03990 (19)	0.04859 (16)	0.14922 (11)	0.0605 (4)	
H14	1.0655 (18)	0.1354 (14)	0.1248 (9)	0.073*	

### Atomic displacement parameters (Å^2^)

**Table:**

	*U*^11^	*U*^22^	*U*^33^	*U*^12^	*U*^13^	*U*^23^
N1	0.0550 (7)	0.0462 (7)	0.0531 (8)	0.0004 (5)	0.0010 (6)	0.0009 (6)
N2	0.0759 (9)	0.0934 (11)	0.0861 (11)	0.0287 (9)	0.0031 (8)	0.0113 (9)
C1	0.0490 (8)	0.0449 (8)	0.0445 (9)	0.0007 (6)	0.0040 (7)	−0.0042 (7)
C2	0.0552 (8)	0.0586 (10)	0.0550 (11)	−0.0002 (8)	0.0104 (8)	0.0092 (8)
C3	0.0482 (8)	0.0703 (11)	0.0606 (11)	0.0040 (8)	0.0109 (8)	0.0044 (9)
C4	0.0509 (8)	0.0485 (9)	0.0473 (9)	0.0033 (7)	−0.0005 (7)	−0.0045 (7)
C5	0.0621 (9)	0.0525 (9)	0.0470 (10)	0.0015 (7)	0.0070 (8)	0.0037 (7)
C6	0.0511 (8)	0.0595 (10)	0.0490 (10)	0.0035 (8)	0.0102 (7)	0.0008 (8)
C7	0.0531 (8)	0.0507 (9)	0.0518 (10)	0.0023 (7)	0.0060 (7)	−0.0034 (8)
C8	0.0597 (9)	0.0658 (11)	0.0571 (11)	0.0072 (9)	0.0036 (8)	−0.0016 (9)
C9	0.0559 (8)	0.0414 (8)	0.0447 (9)	0.0025 (7)	0.0024 (7)	−0.0017 (7)
C10	0.0656 (9)	0.0498 (9)	0.0535 (10)	−0.0058 (8)	−0.0024 (8)	0.0003 (8)
C11	0.0921 (12)	0.0461 (10)	0.0556 (11)	−0.0021 (9)	−0.0072 (9)	0.0045 (8)
C12	0.0790 (12)	0.0624 (11)	0.0565 (11)	0.0186 (9)	−0.0047 (9)	0.0014 (9)
C13	0.0580 (9)	0.0749 (12)	0.0718 (12)	0.0052 (9)	0.0051 (9)	0.0107 (10)
C14	0.0570 (9)	0.0559 (9)	0.0682 (12)	0.0006 (8)	0.0046 (8)	0.0136 (9)

### Geometric parameters (Å, °)

**Table:**

N1—C7	1.2686 (17)	C6—H6	0.967 (12)
N1—C9	1.4177 (16)	C7—H7	0.999 (12)
N2—C8	1.1429 (17)	C9—C10	1.3835 (18)
C1—C6	1.3845 (19)	C9—C14	1.3838 (18)
C1—C2	1.3888 (17)	C10—C11	1.376 (2)
C1—C7	1.4635 (18)	C10—H10	0.971 (12)
C2—C3	1.3683 (19)	C11—C12	1.373 (2)
C2—H2	0.950 (14)	C11—H11	0.949 (15)
C3—C4	1.3883 (19)	C12—C13	1.370 (2)
C3—H3	0.979 (13)	C12—H12	0.970 (14)
C4—C5	1.3823 (19)	C13—C14	1.3801 (19)
C4—C8	1.436 (2)	C13—H13	0.965 (14)
C5—C6	1.3823 (19)	C14—H14	0.975 (14)
C5—H5	0.975 (13)		
			
C7—N1—C9	119.40 (12)	C1—C7—H7	116.7 (8)
C6—C1—C2	118.54 (13)	N2—C8—C4	178.87 (17)
C6—C1—C7	120.26 (12)	C10—C9—C14	119.13 (13)
C2—C1—C7	121.16 (13)	C10—C9—N1	117.48 (12)
C3—C2—C1	120.87 (14)	C14—C9—N1	123.25 (13)
C3—C2—H2	122.3 (8)	C11—C10—C9	120.22 (14)
C1—C2—H2	116.8 (8)	C11—C10—H10	123.0 (8)
C2—C3—C4	119.97 (14)	C9—C10—H10	116.8 (8)
C2—C3—H3	119.9 (8)	C12—C11—C10	120.37 (16)
C4—C3—H3	120.1 (8)	C12—C11—H11	122.7 (9)
C5—C4—C3	120.20 (13)	C10—C11—H11	116.9 (9)
C5—C4—C8	120.42 (13)	C13—C12—C11	119.72 (16)
C3—C4—C8	119.37 (13)	C13—C12—H12	119.9 (8)
C6—C5—C4	119.08 (14)	C11—C12—H12	120.3 (8)
C6—C5—H5	122.3 (7)	C12—C13—C14	120.48 (16)
C4—C5—H5	118.6 (7)	C12—C13—H13	121.9 (9)
C5—C6—C1	121.34 (14)	C14—C13—H13	117.6 (9)
C5—C6—H6	121.2 (8)	C13—C14—C9	120.02 (14)
C1—C6—H6	117.5 (8)	C13—C14—H14	121.0 (8)
N1—C7—C1	121.76 (13)	C9—C14—H14	118.9 (8)
N1—C7—H7	121.6 (7)		
			
C6—C1—C2—C3	−0.7 (2)	C2—C1—C7—N1	−5.6 (2)
C7—C1—C2—C3	176.87 (14)	C7—N1—C9—C10	−146.46 (13)
C1—C2—C3—C4	0.1 (2)	C7—N1—C9—C14	37.8 (2)
C2—C3—C4—C5	0.7 (2)	C14—C9—C10—C11	−2.7 (2)
C2—C3—C4—C8	−178.79 (13)	N1—C9—C10—C11	−178.56 (14)
C3—C4—C5—C6	−0.8 (2)	C9—C10—C11—C12	1.6 (2)
C8—C4—C5—C6	178.69 (13)	C10—C11—C12—C13	0.5 (3)
C4—C5—C6—C1	0.1 (2)	C11—C12—C13—C14	−1.5 (3)
C2—C1—C6—C5	0.6 (2)	C12—C13—C14—C9	0.4 (3)
C7—C1—C6—C5	−176.99 (12)	C10—C9—C14—C13	1.6 (2)
C9—N1—C7—C1	−175.33 (11)	N1—C9—C14—C13	177.28 (14)
C6—C1—C7—N1	172.00 (13)		

### Hydrogen-bond geometry (Å, °)

**Table:**

*D*—H···*A*	*D*—H	H···*A*	*D*···*A*	*D*—H···*A*
C3—H3···N2^i^	0.980 (13)	2.616 (14)	3.473 (2)	146.1 (10)
C5—H5···Cg^ii^	0.975 (13)	2.650 (14)	3.5970 (17)	163.9 (11)

Symmetry codes: (i) −*x*, −*y*+1, −*z*; (ii) *x*−1/2, −*y*+1/2, *z*−1/2.
